# Imaging aids in the diagnosis of reninoma: a case series

**DOI:** 10.1093/ehjcr/ytag012

**Published:** 2026-01-12

**Authors:** Yu Ma, Qian Ge, Yanyan Lin, Pingjin Gao, Jianzhong Xu, Jiguang Wang

**Affiliations:** Department of Cardiovascular Medicine, State Key Laboratory of Medical Genomics, Shanghai Key Laboratory of Hypertension, Shanghai Institute of Hypertension, Shanghai 200025, China; Department of Hypertension, Ruijin Hospital, Shanghai Jiao Tong University School of Medicine, Shanghai 200025, China; Department of Cardiovascular Medicine, State Key Laboratory of Medical Genomics, Shanghai Key Laboratory of Hypertension, Shanghai Institute of Hypertension, Shanghai 200025, China; Department of Hypertension, Ruijin Hospital, Shanghai Jiao Tong University School of Medicine, Shanghai 200025, China; Department of Ultrasound, Ruijin Hospital, Shanghai Jiao Tong University School of Medicine, Shanghai 200025, China; Department of Cardiovascular Medicine, State Key Laboratory of Medical Genomics, Shanghai Key Laboratory of Hypertension, Shanghai Institute of Hypertension, Shanghai 200025, China; Department of Hypertension, Ruijin Hospital, Shanghai Jiao Tong University School of Medicine, Shanghai 200025, China; Department of Cardiovascular Medicine, State Key Laboratory of Medical Genomics, Shanghai Key Laboratory of Hypertension, Shanghai Institute of Hypertension, Shanghai 200025, China; Department of Hypertension, Ruijin Hospital, Shanghai Jiao Tong University School of Medicine, Shanghai 200025, China; Department of Cardiovascular Medicine, State Key Laboratory of Medical Genomics, Shanghai Key Laboratory of Hypertension, Shanghai Institute of Hypertension, Shanghai 200025, China; Department of Hypertension, Ruijin Hospital, Shanghai Jiao Tong University School of Medicine, Shanghai 200025, China

**Keywords:** Reninoma, Hypertension, Hypokalaemia, Contrast-enhanced ultrasound, Case series

## Abstract

**Background:**

Reninoma, a rare juxtaglomerular cell tumour, causes secondary hypertension due to renin hypersecretion. Despite characteristic biochemical features (hypertension, hypokalaemia, and elevated renin–angiotensin–aldosterone system activity), diagnostic challenges persist due to its rarity and phenotypic heterogeneity.

**Case summary:**

We reported two young males with surgically cured hypertension secondary to renin-secreting juxtaglomerular cell tumours. Both presented with refractory hypertension and hypokalaemia. They exhibited discordant renin levels but shared concordant imaging findings. Post-operative pathological immunohistochemistry definitive confirmed reninoma.

**Discussion:**

Our cases highlight the diagnostic challenges of reninoma. In hypertensive patients with hypokalaemia, reninoma should be considered despite its rarity. Normal plasma renin activity cannot definitively exclude reninoma. We recommend plasma renin concentration testing alongside multimodality imaging-contrast-enhanced computed tomography (CT), magnetic resonance imaging, and contrast-enhanced ultrasound to facilitate diagnosis. Both cases were ultimately confirmed by definitive immunohistochemical pathology.

Learning points

- Reninoma may present with normal plasma renin activity (PRA), which should not be used to exclude the diagnosis.
- Contrast-enhanced CT, magnetic resonance imaging (MRI) scans, and contrast-enhanced ultrasound are pivotal modalities for the clinical diagnosis of reninomas.

## Introduction

Reninoma, which is a rare juxtaglomerular cell tumour first described by Robertson *et al.* in 1967,^1^ represents a critical yet underrecognized aetiology of secondary hypertension. Despite its established pathophysiological framework, a total of 272 cases have been reported until October 2025.^2^

Reninoma exerts profound systemic consequences through elevated levels of renin and aldosteronism, which are associated with an increased risk of cardiovascular damage.^3,4^ Consequently, the early detection of reninoma is crucial to maximize the likelihood of achieving a complete cure of hypertension by means of tumour removal, as well as for preventing the onset of resistant hypertension and reducing the risk of long-term cardiovascular and renal complications. However, diagnosing reninomas poses significant challenges due to insufficient understanding of the condition, non-specific symptoms, and atypical laboratory or imaging findings, which often mimic primary aldosteronism or other aetiologies of secondary hypertension. We describe two different cases of reninomas, highlighting the clinical and imaging manifestations and evaluation of this rare disorder.

## Summary figure

**Figure:**
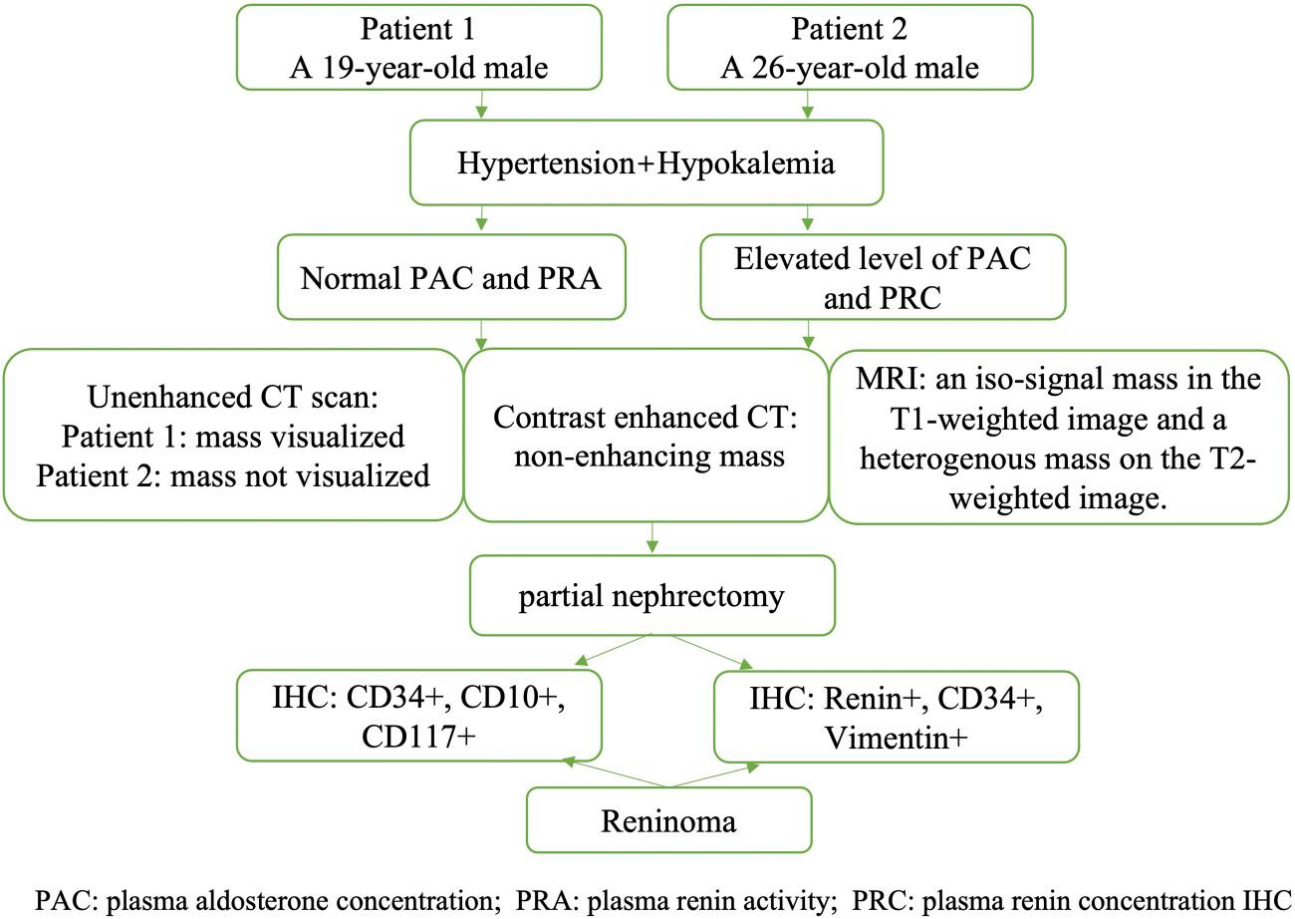


## Case reports

**Figure 1:**
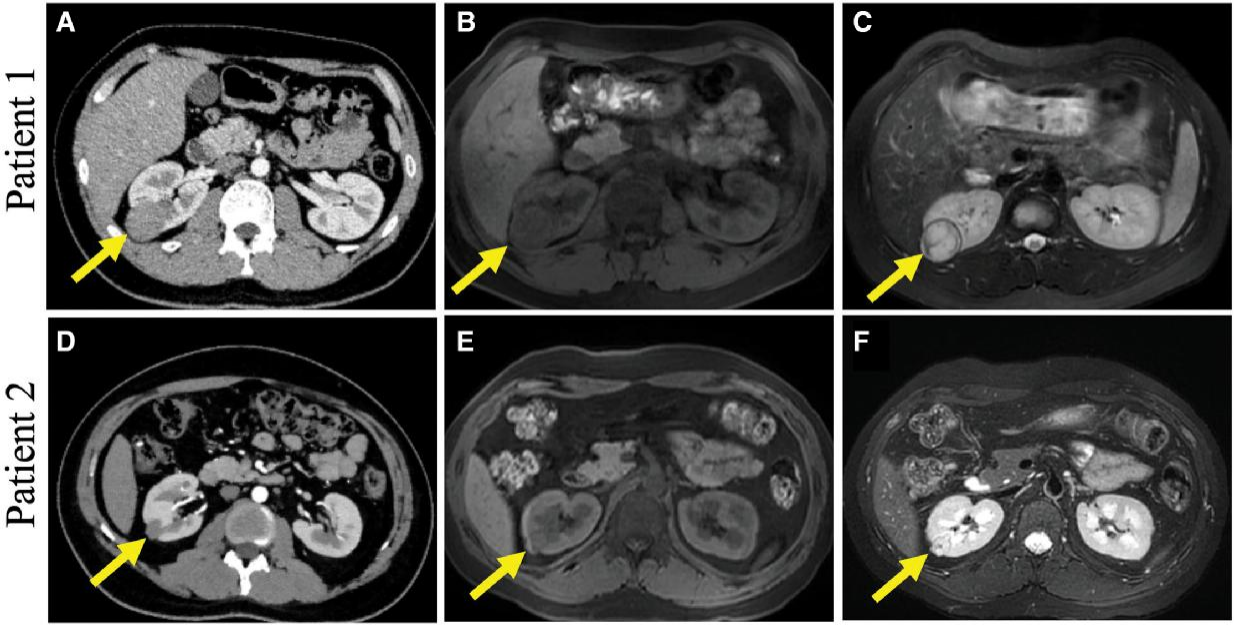
CT and magnetic resonance imaging of two patients. (*A*) Enhanced CT scan showed a round-shaped mass, with no apparent enhancement in the upper pole of the right kidney. (*B–C*) Renal magnetic resonance imaging showed an iso-signal mass in the T1-weighted image and a heterogeneous mass on the T2-weighted image. (*D*) The CT scan showed an irregular nodule in the mid- to back outer side of the right kidney, with no enhancement on the contrast-enhanced scan (arrow). (*E–F*) The mass showed iso-signal intensity and a heterogeneous signal intensity in the T1- and T2-weighted magnetic resonance imaging.

Patient 1, a 19-year-old male patient presented with a 2-year history of severe hypertension and hypokalaemia. He had been treated with amlodipine besylate tablets and sustained-release potassium chloride. He was referred to our department for evaluation of secondary hypertension. Notably, he did not exhibit symptoms such as headache or fatigue. On physical examination, his blood pressure (BP) was 145/105 mmHg. Laboratory analyses showed hypokalaemia with serum potassium being 3.15 mmol/L (reference: 3.5–5.1 mmol/L) and high 24-h urine aldosterone excretion at 21.97 ug/24 h (reference: 2.36–18.36 ug/24 h). However, the plasma aldosterone concentration (PAC, 222.02 pg/mL，reference: 29.06–332.78 pg/mL) and plasma renin activity (PRA, 1.45 ng/mL/h, reference: 0.73–17.4 ng/mL/h) was normal in the standing position. Abdominal ultrasound identified a 3.3 × 3.2 cm mass in the right kidney characterized by clear margins, absence of internal calcification, and lack of posterior acoustic shadowing, suggestive of a renal hamartoma. Subsequently, computed tomographic angiogram (CTA) showed normal renal arteries and slight thickening of the left adrenal gland without significant nodules or adenomas. Additionally, a round, non-enhancing mass was detected in the upper pole of the right kidney (*Figure 1A*). Renal magnetic resonance imaging (MRI) showed an iso-signal mass in the T1-weighted image (*Figure 1B*) and a heterogeneous mass in the T2-weighted image (*Figure 1C*)

**Figure 2:**
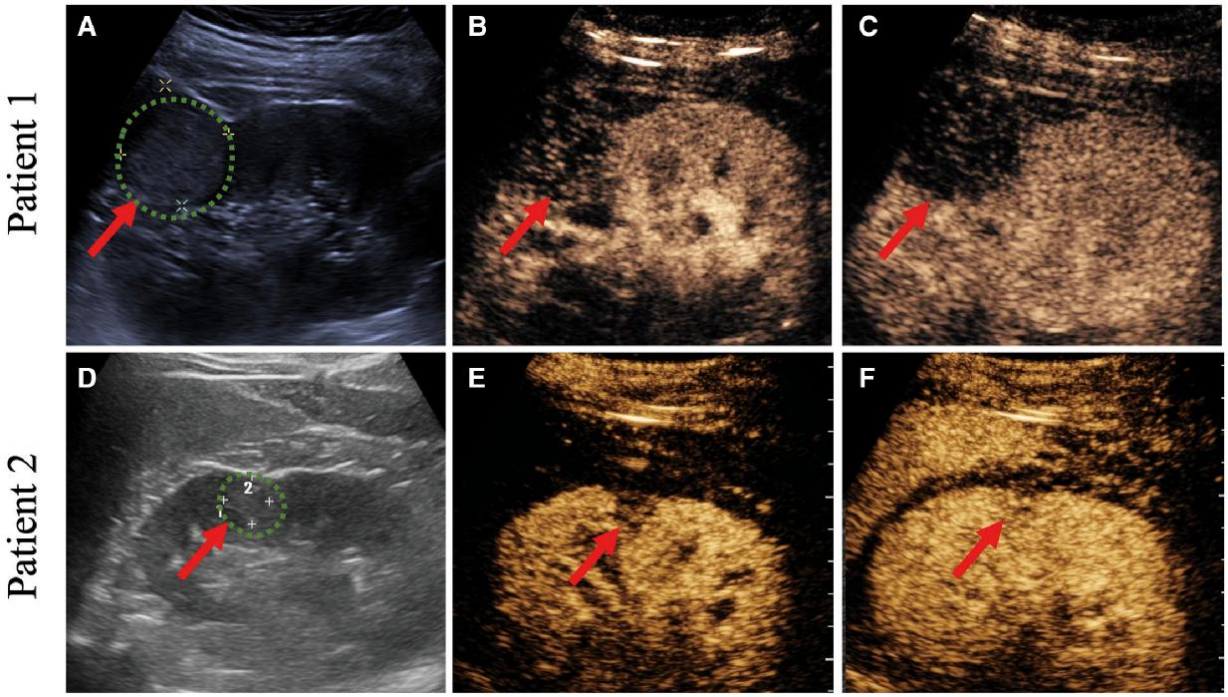
Contrast-enhanced ultrasound of two patients. (*A*) Greyscale ultrasound showed a circular, slightly hyperechoic mass measuring 32 × 35 mm in the mid-to-upper pole of the right kidney. (*B–C*) Contrast-enhanced ultrasound showed hypoperfusion within the mass in the cortical (*B*) and medulla phase (*C*). (*D*) Greyscale ultrasound showed a hypoechoic lesion in the upper pole of the kidney, ∼14 × 15 mm in size. (*E–F*) Contrast-enhanced ultrasound showed hypoperfusion within the lesion in the cortical (*E*) and medulla phase (*F*).

For further examination, contrast-enhanced ultrasound (CEUS) revealed that after contrast agent injection, the wash-in in the mass was slightly later than in the renal parenchyma, while the wash-out was earlier than the renal parenchyma, and the perfusion peak value was significantly lower than in the renal parenchyma, with uneven perfusion (*Figure 2A–C*). Although this lesion is likely benign, its appearance is atypical. While the greyscale ultrasound features resemble renal angiomyolipoma (slightly hyperechoic), the contrast-enhanced ultrasound perfusion pattern differs from the progressive enhancement typical of angiomyolipoma. Furthermore, its contrast-enhanced ultrasound appearance does not match the hypervascular pattern typical of most renal cell carcinoma (RCC), nor the characteristic uniform perfusion of eosinophilic adenoma. However, given the patient’s clinical symptoms, reninoma emerges as the primary diagnostic consideration.

**Figure 3:**
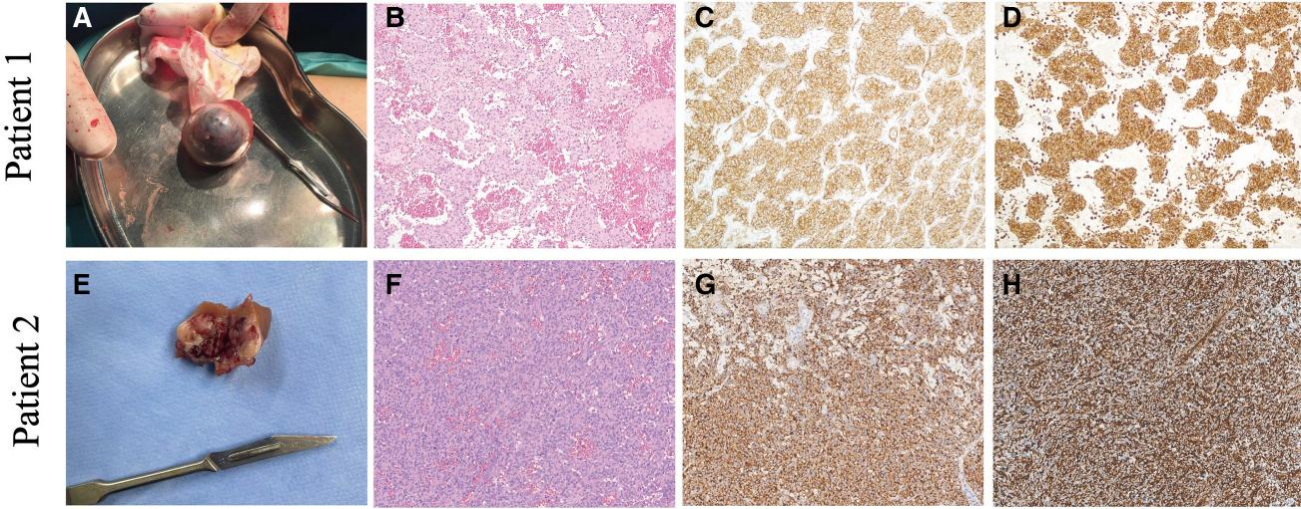
Histologic features of the tumours. (*A–D*) Patient1. (*A*) The gross appearance of a solitary and well-encapsulated mass. (*B*) Haematoxylin and eosin staining of the tumour (10×). (*C*) Tumour cells were positive for CD34 (10×). (*D*) Tumour cells were positive for vimentin (10×). (*E–H*) Patient 2. (*E*) The gross appearance of a cross-section of the partial nephrectomy specimen, with a mass on the cut surface. (*F*) Haematoxylin and eosin staining of the tumour (10×). (*G*) Tumour cells were positive for renin (10×). (*H*) Tumour cells were positive for CD34 (10×).

Considering that the mass was relatively large, and the possibility of malignancy could not be ruled out, surgery was performed. The gross examination demonstrated a solitary and well-encapsulated mass (*Figure 3A*). Haematoxylin and eosin (H&E) staining demonstrated sheets of tumour cells arranged in a lobular growth pattern, interspersed with dilated and congested small blood vessels within the tumour stroma (*Figure 3B*). Immunohistochemical stains showed the tumour cells to be positive for CD34 (*Figure 3C*), vimentin (*Figure 3D*), CD10, and CD117. One week after the surgery, the patient was normotensive without any medication.

Patient 2, a 26-year-old man was referred to our hospital due to hypertension, frequent headache, nocturia, and fatigue. He had a 6-year history of hypertension and was treated with losartan potassium and hydrochlorothiazide tablets, at a dose of 50 mg/12.5 mg daily. His father had a history of hypertension.

On physical examination, his BP was 140/100 mmHg, and laboratory examinations showed hypokalaemia (serum K + 2.99 mmol/L, reference: 3.5–5.1 mmol/L), elevated level of PAC, and plasma renin concentration (PRC). Considering that potassium level may be affected by losartan potassium and hydrochlorothiazide, after changing losartan and hydrochlorothiazide for amlodipine besylate and diltiazem hydrochloride for 4 weeks, the patient was admitted to our department.

On admission, his BP and heart rate were 166/110 mmHg and 72 beats/min, respectively. The laboratory findings indicated hypokalaemia, with a serum potassium level of 2.59 mmol/L. Additionally, PAC (457.36 pg/mL, reference: 29.06–332.78 pg/mL) and PRC (500 pg/mL, reference: 4–38 pg/mL) were markedly increased in the upright position. The abdominal ultrasound was normal, and a computed tomography (CT) scan without contrast showed no obvious abnormalities. However, a 1.4 × 1.5 cm non-enhancing mass was detected in the mid- to back outer side of the right kidney on the contrast-enhanced CT scan (*Figure 1D*). Renal artery stenosis was excluded by ultrasound with Doppler and contrast-enhanced CT scan. Magnetic resonance imaging also revealed a small solid mass in the same right kidney, which had iso-signal intensity in the T1-weighted image (*Figure 1E*) and a heterogeneous signal intensity in the T2-weighted image (*Figure 1F*). The tumour was also visible on CEUS. After injection of ultrasound contrast agent sulfur hexafluoride microbubbles, beginning of the perfusion of nodules in the arterial phase was slightly later than that of the surrounding renal parenchyma, showing a slightly lower enhancement. After the parenchymal phase, it showed lower enhancement compared with the surrounding renal parenchyma (*Figure 2D–F*). This manifestation is inconsistent with the common high perfusion of clear cell RCC and is also inconsistent with the uneven perfusion of papillary RCC. Although distinction from oncocytic adenoma can be challenging, the latter typically manifests as a large lesion with uniform echogenicity and homogeneous perfusion—features absent in this patient. Together with the patient’s medical history, the ultrasound contrast findings suggest a benign right renal mass, for which reninoma remains a diagnostic consideration.

Following partial nephrectomy, the patient’s BP measured 130/80 mmHg without the use of medications at the 1-month post-operative visit. Plasma aldosterone concentration and PRC were within normal limits (<14.5 pg/mL and 36.5 pg/mL, respectively). Histopathological examination confirmed a reninoma. The partial nephrectomy specimen measured 2.5 × 2.4 × 2.4 cm. Gross examination revealed a moderately well-demarcated tumour, 1.7 cm diameter tumour within the renal cortex, which exhibited a variegated grey–white to tan cut surface and firm consistency (*Figure 3E*). Haematoxylin and eosin staining demonstrated nests of tumour cells arranged in a nodular pattern, accompanied by prominent microvascular proliferation within the stromal compartment (*Figure 3F*). Immunohistochemically, the tumour cells showed reactivity with antibodies to renin (*Figure 3G*), CD34 (*Figure 3H*), and vimentin.

## Discussion

We present two cases that were diagnosed at our department over 9 years, displaying different diagnostic challenges.

Hypertension and hypokalaemia were present in both cases. Patient 1 had no symptoms other than hypertension and hypokalaemia, whereas Patient 2 presented with headaches. According to the previous reports, the most common initial symptom of reninoma is headache, followed by dizziness, nocturia, polyuria, and vomiting.^5–7^ However, all these symptoms are non-specific.

In both cases, the tumour was localized within the right kidney. The tumour in Patient 1 was relatively large and protruded from the surface of the kidney, while the tumour in Patient 2 was endogenic, with no obvious external process on the surface of the kidney. Conventional ultrasound and unenhanced CT scan missed the mass in Patient 2. For renin results, there is a significant difference between the two cases. The PRA was normal in Patient 1, whereas the PRC was markedly elevated in Patient 2 (the results were repeated twice in all cases). To our knowledge, renin levels have been reported mildly to severely elevated in previous cases. Previous studies reported that the mean PRA was 12 times above the upper limit of normal (ULN), and the median PRA was also 12 times above the ULN.^8–10^ Our case (Patient 1) indicated that reninoma can present as normal renin levels, which is different from our previous understanding about reninoma.

Renin is an enzyme with biological activity, and its function is to cleave angiotensinogen into angiotensin I.^11^ Plasma renin activity measures the rate at which angiotensin I is generated from angiotensinogen within a specific time period, reflecting the actual catalytic activity of renin in the plasma. Therefore, the activity of renin is influenced by the concentration of angiotensinogen and the interaction between renin and its substrate, and it may not reflect the actual concentration of active renin. Plasma renin concentration is measured using a chemiluminescent immunoassay, which directly measures the number of renin molecules in the plasma using specific antibodies. Even if the active form of renin is diminished, the total renin concentration may still be elevated due to excessive secretion from the tumour. Therefore, for patients suspected of having a reninoma, it is recommended to measure PRC rather than PRA, as testing PRA may lead to missed or misdiagnosis.

In addition to laboratory data, imaging tools are important to diagnose and localize the renal lesions. Enhanced CT or MRI, along with contrast-enhanced ultrasound, is the primary choice for identifying reninomas. In our cases, lesions showed an iso-density area on plain CT and a low-density area on enhanced CT. Magnetic resonance imaging shows reninomas as iso-signal intensity on the T1-weighted images and as heterogeneous intensity on the T2-weighted images. Despite divergent conventional sonographic features between the two cases, a consistent contrast-enhancement signature was observed on CEUS: the renal masses exhibited delayed contrast arrival during the arterial phase, with region-of-interest measurements confirming persistently lower peak enhancement intensity throughout all vascular phases. These findings may serve as a critical basis for distinguishing this type of tumour from common RCCs and angiomyolipoma.

Our experience underscores specific clinical scenarios that should raise suspicion for reninoma. This diagnosis should be considered in young or middle-aged patients, particularly those without a family history of hypertension, who present with treatment-resistant or early-onset hypertension accompanied by unexplained hypokalaemia. Importantly, as demonstrated in our first case, normal PRA does not definitively exclude the diagnosis. In such cases, measurement of PRC is recommended. When clinical and biochemical suspicion exists, multimodality imaging is crucial. Contrast-enhanced ultrasound plays a particularly valuable role in this context. As illustrated in both our cases, reninomas consistently demonstrated a characteristic CEUS pattern: delayed arterial-phase enhancement (wash-in) followed by rapid wash-out, resulting in a peak enhancement intensity that was consistently lower than that of the surrounding renal parenchyma. This ‘hypovascular’ pattern is a key feature that helps differentiate reninomas from the hypervascular appearance of clear cell RCC and the progressive enhancement of angiomyolipoma, thereby aiding in pre-operative diagnosis. The differential diagnosis for a small renal mass in a hypertensive patient includes other secondary hypertension aetiologies such as aldosterone-producing adenomas, as well as more common renal tumours. The principal challenge lies in the tumour’s rarity and its phenotypic mimicry of other conditions. For instance, its imaging characteristics can overlap with other hypovascular tumours such as papillary RCC or oncocytoma.

Of course, the gold standard for the diagnosis of reninoma is pathology. In addition to renin levels, the diagnosis of reninoma can also rely on non-invasive examinations including CEUS, enhanced CT, and MRI. Provision of clinical, laboratory, and imaging manifestations will help to increase awareness of reninoma as the probable cause of hypertension and hypokalaemia, even in the presence of normal renin levels.

## Lead author biography


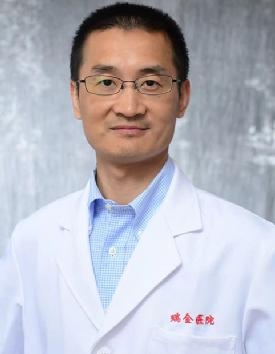


Dr Jianzhong Xu is a senior cardiologist specializing in hypertension and coronary artery disease. He works at the Department of Hypertension, Ruijin Hospital, Shanghai Jiao Tong University School of Medicine, Shanghai, China. Dr Xu conducted research as a visiting scholar at the Baker Heart Research Institute and Alfred Hospital in Melbourne, Australia.

## Data Availability

The data underlying this article are available from the corresponding author.
